# Editorial: Memories for the future

**DOI:** 10.3389/fpsyg.2024.1502718

**Published:** 2024-10-15

**Authors:** Markus Boeckle, Ueli Kramer, Nicola S. Clayton

**Affiliations:** ^1^Karl Landsteiner University of Health Sciences, Krems, Austria; ^2^Division of Psychiatry and Psychotherapeutic Medicine, University Hospital Tulln, Tulln, Austria; ^3^University Institute of Psychotherapy, Centre Hospitalier Universitaire Vaudois, Lausanne, Switzerland; ^4^Department of Psychology, University of Cambridge, Cambridge, United Kingdom

**Keywords:** episodic memory, future-oriented behavior, memory reconsolidation, future planning, animal cognition, psychotherapy, cognitive psychology, behavioral neuroscience

Our understanding of episodic memory has evolved significantly, revealing its critical role in shaping decision-making for current and future events. The Research Topic of “*Memories for the future*,” edited by Markus Boeckle, Nicky Clayton, and Ueli Kramer, spans theoretical work, pedagogical tools, and practical applications in psychotherapy and political decision-making.

Human memory is a complex system comprising various subsystems, including working, episodic, semantic, perceptual, and procedural memory (Finotelli and Eustache). These interconnected systems allow humans—and, as research shows, some non-human animals—to recall past events and navigate future challenges (Schacter and Addis, 2007; Boeckle and Clayton, 2017; Clayton et al., 2003). The plasticity of episodic memories enables them to change during recall, making them powerful tools for adapting behavior and improving mental health, particularly in psychotherapy (Biderman and Shohamy, 2021; Bordalo et al., 2024; Ecker, 2024; Ecker and Vaz, 2022).

The malleability of episodic memory is central to therapeutic approaches like memory reconsolidation, where reactivated memories are altered before being stored again, leading to lasting therapeutic changes (Ecker and Vaz, 2022; Lane et al., 2015). Studies have shown that guided recall of positive autobiographical memories can enhance psychological resources, reduce depressive symptoms, and increase anticipated pleasure, highlighting memory's profound impact on mental wellbeing (Hallford et al., 2024, Raeder et al.). Additionally, Ecker (2024) proposes that most psychotherapeutic actions can be understood as memory modification processes, further emphasizing the centrality of memory in therapeutic change. As Kramer (2021) highlighted in his review of Lane and Nadel's book “*Neuroscience of enduring change*” (Lane and Nadel, 2020), there is a crucial need for further studies that explore the interaction between memory change and emotional change within psychotherapy.

Research on episodic-like memory in animals further challenges the traditional view that complex memory systems are exclusive to humans. Studies on species like corvids and great apes show that they can recall past events to plan for future needs, offering comparative insights that deepen our understanding of human and animal cognition (Boeckle and Bugnyar, 2012; Boeckle and Clayton, 2017; Boeckle et al., 2018; Clayton et al., 2003; Gruber et al., 2019; Miller et al., 2020; Osvath and Martin-Ordas, 2014; Clayton and Dickinson, 1998; Raby and Clayton, 2009; Raby et al., 2007). By studying how animals use episodic-like memory, researchers can uncover fundamental principles that illuminate memory's evolutionary roots (Rossi et al., 2021).

This Research Topic on “*Memories for the future*” brings together diverse research contributions, fostering interdisciplinary collaboration and inviting exploration of how memories, shaped by past experiences, influence behavior. This Research Topic aims to develop new theoretical frameworks and innovative therapeutic techniques, bridging the gap between basic research and clinical application.

The article “*Effects of memory cue and interest in remembering and forgetting of gist and details*” (Hu and Yang) explores how memory cues and personal interest affect the retention and forgetting of gist and detailed information. The researchers found that memory cues significantly influence the forgetting rates of gist and details. Specifically, when gist cues were used, gist memories were retained longer than detailed ones, and vice versa. Interestingly, while subjective interest improved memory accuracy, it did not impact the forgetting rates of gist and details. The study highlights the complex interplay between external cues and intrinsic motivation in memory processes, suggesting potential strategies for enhancing memory retention.

The meta-analysis about narrative-based autobiographical memory interventions (NBI) by Raeder et al. indicates that NBIs, which involve restructuring a patient's narrative to create a coherent and positive life story, are effective in reducing PTSD symptoms. The study underscores the importance of memory reconsolidation in these interventions, where reactivated memories are modified to achieve therapeutic benefits. The findings suggest that NBIs provide long-lasting symptom relief and are more effective than non-NBIs in addressing PTSD, highlighting their potential as a therapeutic tool.

The study by Zeng et al. examines how episodic memory contributes to spatial learning, highlighting its role in different learning paradigms: one-shot learning, replay learning, and online learning. The research shows that episodic memory enhances learning efficiency, particularly in complex tasks requiring rapid adaptation. The findings emphasize that replay learning, which mimics natural memory processes like those in the hippocampus, leads to better long-term performance than one-shot or online learning. These results underscore the critical role of episodic memory in navigating and solving spatial challenges, offering insights into its broader implications for understanding cognitive processes and adaptive behavior.

Tanguay et al. explore the effectiveness of episodic specificity induction (ESI) in enhancing children's episodic future thinking (EFT). The research investigates whether prompting children to vividly imagine future events can improve their performance on tasks requiring future-oriented cognition. The findings indicate that while ESI successfully promoted the construction of detailed future events, the overall effect on children's performance was modest, suggesting that the cognitive demands of such interventions might be challenging for young children. The implications of this study are significant for understanding how EFT can be nurtured in developmental stages, albeit with potential limitations due to cognitive maturity.

The review of various mathematical models by Finotelli and Eustache describes human memory, focusing on working, episodic, semantic, perceptual, and procedural memory. The authors compare memory process models, highlighting each approach's strengths and limitations. The paper emphasizes the need for further development to better understand memory's role in cognition and its disorders. This work contributes significantly to the ongoing effort to model human memory mathematically, offering a foundation for future research in cognitive science and neuropsychology.

The potential of memory research to transform our understanding of human cognition is immense, offering insights into political decision-making, psychotherapeutic interventions, and educational tools. As we unravel the complexities of memory processes in humans and animals, we gain a deeper understanding of how memory shapes interactions with time, space, and the environment. The “*Memories for the future*” issue underscores this dynamic interplay between memory and behavior, highlighting its critical role in shaping our world and the transformative potential of memory-based therapies. This research is foundational to advancing cognition studies and fostering psychological wellbeing.
